# Evaluation of Antibiotic Resistance in Bacterial Strains Isolated from Sewage of Slaughterhouses Located in Sicily (Italy)

**DOI:** 10.3390/ijerph18189611

**Published:** 2021-09-12

**Authors:** Alessio Facciolà, Antonino Virga, Maria Eufemia Gioffrè, Pasqualina Laganà

**Affiliations:** 1 Department of Biomedical and Dental Sciences and Morphofunctional Imaging, University of Messina, 98125 Messina, Italy; afacciola@unime.it (A.F.); emi.gioffre@alice.it (M.E.G.); 2 Department of Chemical, Biological, Pharmaceutical and Environmental Sciences, University of Messina, 98166 Messina, Italy; antoninovirga58@gmail.com

**Keywords:** wastewater, slaughterhouses, antibiotic resistance

## Abstract

Antimicrobial resistance is presently one of the most public health critical concerns. The frequent and often incorrect use of antibiotics in animal husbandry has led to the spread of antimicrobial resistance in this setting. Wastewater from slaughterhouses can be contaminated with multidrug-resistant bacteria, representing a possible cross-contamination route. We evaluated the presence of antibiotic-resistant bacteria in wastewater samples from slaughterhouses located in an Italian region. Specifically, 18 slaughterhouses were included in the study. Of the tested samples, 40 bacterial strains were chosen, identified, and tested for antibiotic susceptibility. *Pseudomonas* spp., *Proteus* spp., *Enterobacter* spp., *Aeromonas* spp., and *Citrobacter* spp. were the most detected genera. The most resistant strains were on average those belonging to *Enterobacter* spp. The highest resistance rate was recorded for macrolides. Among β-lactams, penicillins and cephalosporins were by far the molecules towards which the highest resistance was detected. A very interesting finding is the difference found in strains detected in wastewater from poultry slaughterhouses, in which higher levels for almost all the considered drugs were detected compared to those from ungulates slaughterhouses. Our results indicate wastewater from slaughterhouses as a potential vehicle of resistant bacteria and highlight the importance of correct management of these kinds of waters.

## 1. Introduction

Global development and spread of antimicrobial resistance (AMR) have become a crucial concern in public health [1]. In some countries, antibiotic resistance has been so widespread that some bacterial strains are resistant to nearly all of the drugs used [2]. In order to counteract this hazard, the World Health Organization (WHO) developed a prioritization list of the most important antimicrobial-resistant bacteria to address the research and development of novel drugs [3]. Within this list, the group of the so-called ESKAPE bacteria (*Enterococcus* spp., *Staphylococcus aureus*, *Klebsiella pneumoniae*, *Acinetobacter baumannii*, *Pseudomonas aeruginosa*, *Enterobacter* spp.), as well as *Escherichia coli*, received the greatest attention as causes of life-threatening infections in healthcare settings worldwide [4,5,6,7].

Antimicrobial overuse occurs in multiple sectors including human, animal, and agricultural settings [8,9]. In particulars, the common and often inappropriate use of antibiotics as therapeutics and growth promoters in animal husbandry has led to the increase in and spread of antimicrobial resistance in livestock-associated bacteria with even the development of novel resistances (e.g., mobile colistin resistance genes) and of cross-resistances to drugs routinely used in human medicine with possible antibiotic treatment failure [10,11,12,13]. To contrast the phenomenon of antibiotic resistance, the use of various natural molecules has also been proposed, such as honey, foods that contain polyphenols, citrus essential oils, etc. [14,15,16].

In recent years, it has been shown that livestock animals are carriers of ESKAPE bacteria and ESBL-producing *E. coli* and can spread to humans by direct contact and/or cross-contamination of food products [17,18]. Moreover, animals can also spread antimicrobial-resistant bacteria to the general environment and excrete and introduce them into slaughterhouses [19,20,21]. In this setting, the process water used at different steps in the slaughtering process (e.g., evisceration) and often contaminated with various multidrug-resistant (MDR) bacteria, can represent a possible cross-contamination route, as shown for poultry slaughterhouses [22]. For these reasons, slaughterhouse wastewater is considered a vehicle for antibiotic-resistant bacteria and thus can play an important role in their environmental spread. Generally, slaughterhouses are equipped with wastewater treatment plants (WWTPs), and then, after the treatment, they discharge their wastewater either into a municipal WWTP or directly into a water body (e.g., river). This process can represent a reservoir for the spread of antimicrobial-resistant bacteria with clinical relevance into the environment and then in the general population with an impact on colonization probability and/or infection caused by ESKAPE bacteria [23,24,25].

In addition, some previous studies have shown that the occupational exposure of farmers and slaughterhouse workers to animals and/or contaminated working environments can also represent an elevated risk to acquired and spread antibiotic-resistant bacteria [26,27,28].

In the European Union, the Decision 2013/652/EU established detailed measures for the harmonized monitoring and reporting of data concerning antimicrobial resistance of zoonotic and commensal bacteria applicable until 31 December 2020. This decision has now been replaced by the Decision 2020/1729/EU, valid from 1 January 2021. The latter, in continuity with the principles and objectives already expressed and obtained with the previous one, aims to continue to obtain comparable and reliable data on antimicrobial resistance in the European Union. Under this decision, in Italy, a similar Harmonized Monitoring Plan on Antimicrobial Resistance of Zoonotic and Commensal Bacteria has been recently enacted in order to obtain data on the prevalence of resistance in bacterial strains included in the plan that are comparable between European Member States [29].

The epidemiological surveillance of food-borne diseases in Europe makes use of the “Rapid Alert System for Food and Feed” (RASFF), a system for the rapid exchange of information between Member States, created in 1979 within the European Community. This system makes it possible to notify, in real time, the risks to human, animal and environmental health from “dangers” present in food intended for human consumption and animal feed. All information is introduced and exchanged between Member States through the specific online platform. One of the reasons for the reports is precisely the finding of antibiotic residues in food and feed. In January 2021, RASFF received a report of foods containing antibiotic residues, notified by France, concerning beef and related imported meat products. In July 2021, Belgium was notified of the finding of a batch of feed additives containing antibiotic resistance genes. In 2020, another batch was notified by Austria due to the presence of antibiotic residues, also in feed [30].

The aim of this study was to evaluate the presence of antibiotic-resistant bacteria in wastewater collected from some slaughterhouses present in the territory of Sicily, a region of southern Italy, in order to highlight the real risk represented by these important settings in the spread and diffusion of antibiotic resistance.

## 2. Materials and Methods

### 2.1. Sampling Area

**Figure 1:**
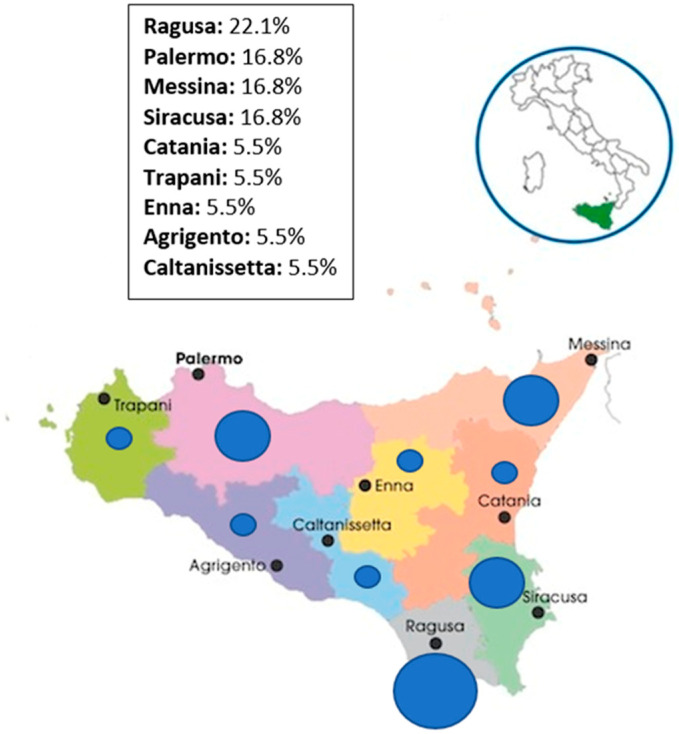
Geographical distribution of the enrolled slaughterhouses and origin percentages of the tested samples.

The sampling activities were conducted from February to June 2021. Of a total of 35 active slaughterhouses distributed throughout the territory of the Sicilian Region, a representative group of 18 (51.4%), located in all nine Sicilian provincial districts, were included in the study. Specifically, we decided to test only the biggest and the most active slaughterhouses of which the most part (88.9%) is intended for the slaughter of domestic ungulates (cattle, pigs, horses, sheep, and goats) while 11.1% slaughters poultry. One sample was detected from each slaughterhouse. A map showing the exact regional origin of the tested samples is provided in Figure 1.

### 2.2. Samples Collection and Treatment

**Figure 2:**
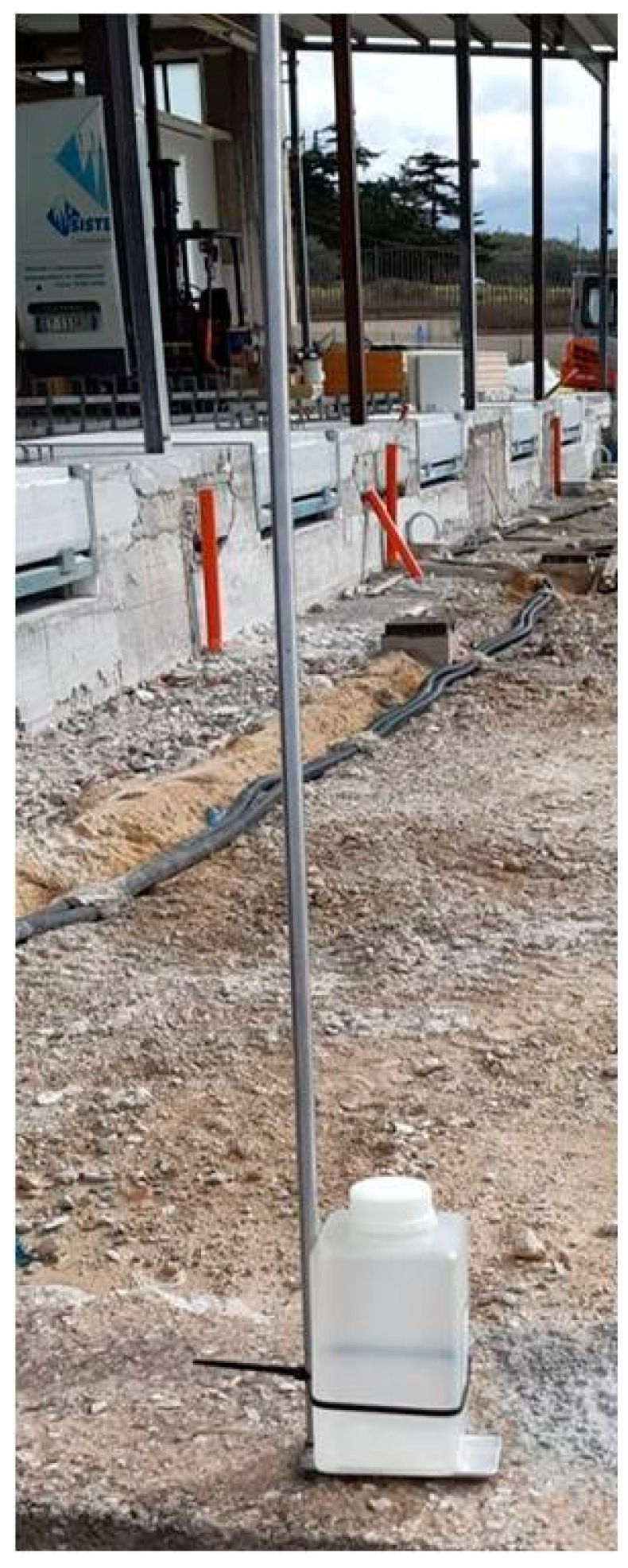
Photo of the stick and sterile container used for sampling.

In each slaughterhouse, the samples were collected at the initial point of the wastewater system before they underwent any treatment. The sampling was performed by properly trained veterinary staff with the help of a long stick, at the end of which there was a support for a 1 L sterile container (Figure 2).

After immersion, the container was disinfected on the outside, labeled, and promptly transported to the laboratory in a cooler kept at 5 ± 2 °C. To remove coarse particles, the samples were manually filtered with the help of stomacher filter bags with a cloth filter (0.5 mm pore size) and subsequently cultured within 24 h of sampling.

### 2.3. Isolation and Selection of Bacteria

After filtration, 20 µL of the samples were directly inoculated on Hektoen Enteric Agar (HEA) for an initial estimate of the Enterobacteriaceae presence and 200 µL were placed in a test tube containing an enrichment medium used for the detection of bile-tolerant Gram-negative bacteria (EE Broth-Mossel). The quantity of sample deposited directly on the HEA medium (20 µL) was chosen after carrying out tests with sowing different quantities (unpublished data) and understanding that a higher quantity of sample would have developed too many overlapping colonies and that with a smaller quantity there was a risk of decreasing its variability. During the isolation, particular care was taken in the choice of colonies among those that presented different phenotypic characteristics (e.g., morphology, size, and pigmentation). Some of these have been taken, in order to obtain as much as possible a collection of strains representative of the community of cultivable *Enterobacteriaceae* present in each sample.

### 2.4. Phenotypic Identification of Bacteria

The isolates were identified to the species or genus level by API 20 NE, API 20 E profiles (bioMerieux, Marcy l’Etoile, France), according to the manufacturer’s instructions.

### 2.5. Antibiotic Susceptibility

Among the isolated microorganisms, some of them have been chosen, based on their greater frequency of isolation and taking care to select enterobacteria from all the samples. Selected strains were tested for resistance or sensitivity to different antibiotics using the standard disk diffusion method (Kirby Bauer test) and commercially available antibacterial disks. For the disk diffusion assay, bacteria were grown at 37 °C for 24 h on plates of Tryptic Soy Agar, harvested, and then suspended in sterile water adjusted to a 0.5 McFarland turbidity standard (bioMerieux), corresponding to 1.5 × 10^8^ CFU mL^−1^. Suspensions were inoculated in triplicate on plates of Müeller–Hinton agar using a cotton swab. After 24 h of incubation at 37 °C, the diameter of inhibition zones was measured with a precision caliper (Mitutoyo, Andover, UK). Each bacterial species was classified as resistant (R), intermediately resistant (I), or sensitive (S) according to the breakpoints established by EUCAST (2021) [31]. All media and antibacterial disks for Kirby Bauer test were produced by ThermoFisher Scientific, Massachusetts, US.

Shown below are 31 different antibiotics used (synthesized as described in Supplementary Materials), grouped into the following classes according to their mechanisms of action [32].

Cell wall inhibiting and disrupting membrane antibiotics:

(1) Beta-lactams, including penicillins (ampicillin (AMP, 10 μg), carbenicillin (CAR, 100 μg), mezlocillin (MEZ, 75 μg), piperacillin (PRL, 100 μg)), monobactams (aztreonam (ATM, 30 μg)), cephalosporins (cefazolin (KZ, 30 μg), cefoxitin (FOX, 30 μg), cefuroxime (CXM, 30 μg), cefotaxime (CTX, 30 μg), ceftazidime (CAZ, 30 μg), ceftriaxone (CRO, 30 μg)), carbapenems (imipinem (IMI,10 μg);
(2) Fosfomycin (FOS, 50 μg);
(3) Polymyxin (colistin sulphate (CS, 10 μg)).

Nucleic acid inhibitors:

(1) Quinolones (nalidixic acid (NA, 30 μg), pipemidic acid (PI, 20 μg)); fluoroquinolones (ciprofloxacin (CIP, 5 μg), levofloxacin (LEV, 5 μg), norfloxacin (NOR, 10 μg) ofloxacin (OFX, 5 μg));
(2) DNA inhibitors (nitrofurantoin (F, 300 μg));
(3) RNA synthesis inhibitors: rifampicins (rifampicin (RD, 30 μg)).

Protein synthesis inhibitors:

(1) Aminoglycosides (amikacin (AK, 30 μg), gentamycin (CN, 10 μg), netilmicin (NET, 30 μg), tobramycin (TOB, 10 μg));
(2) Glicilglicines (tigecycline (TGC, 15 μg));
(3) Macrolides (azithromycin (AZM, 15 μg));
(4) Phenicol derivatives (chloramphenicol (C, 30 μg));
(5) Tetracyclines (tetracycline (TE, 30 μg, CT0054B)).

In addition, the association between an inhibitor of the beta-lactamase and a semi-synthetic penicillin (amoxicillin + clavulanic acid (AUG, 30 μg)) was assayed.

## 3. Results

### 3.1. Bacteriological Monitoring

**Figure 3:**
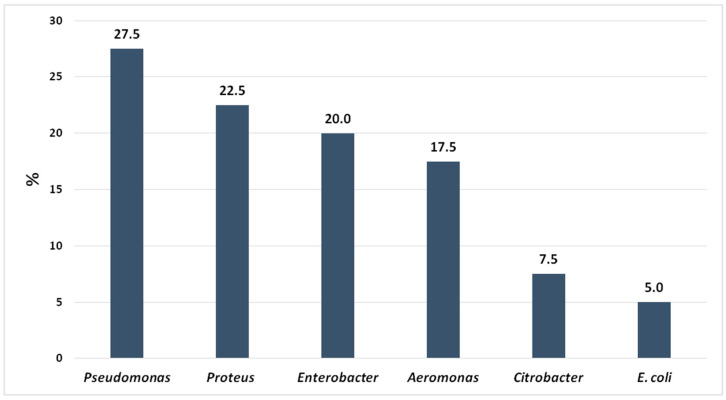
Percentages of the bacterial genera and *E. coli* detected in wastewater samples.

As expected, microorganisms belonging to the Enterobacteriaceae family and other germs typically found in sewage were isolated from all the analyzed samples. The percentages of the detected bacterial genera are shown in Figure 3.

*Pseudomonas* spp. was one of the most commonly detected genera, representing almost a third of all the detected bacteria. Among *Pseudomonas* spp. and *Proteus* spp., *P. aeruginosa* and *P. mirabilis* were the most frequently isolated microorganisms (27.3% and 85.7%, respectively), while *Enterobacter cloacae*, *Aeromonas hydrophila,* and *Citrobacter freundii* were almost all of their respective genera (over 95%).

### 3.2. Evaluation of Antibiotic Resistance

**Figure 4:**
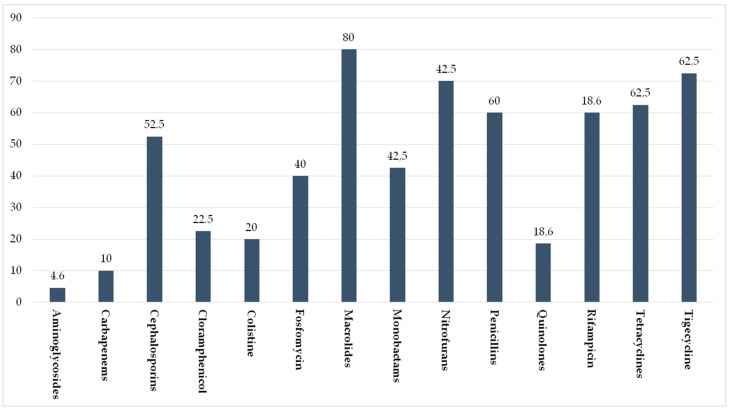
General percentage resistance of the detected bacterial strains to the used antibiotic families.

On the tested samples, 40 bacterial strains were chosen and tested for antibiotic susceptibility. Figure 4 shows the general percentage resistance of the chosen bacterial strains to the used antibiotic families while Table 1 shows the resistance levels per single bacterial genus.

**Figure 5:**
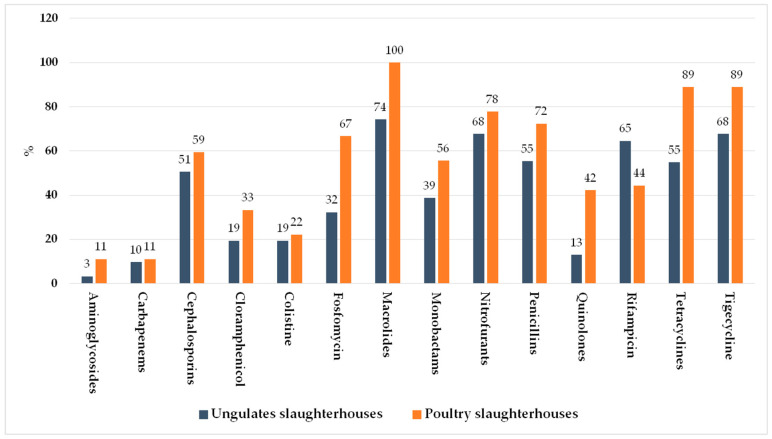
Antibiotic resistance of the strains detected in wastewater from the two different types of studied slaughterhouses (ungulates and poultry).

As reported in Figure 5, there was a remarkable difference in antibiotic resistance between the two different types of slaughterhouses.

**Figure 6:**
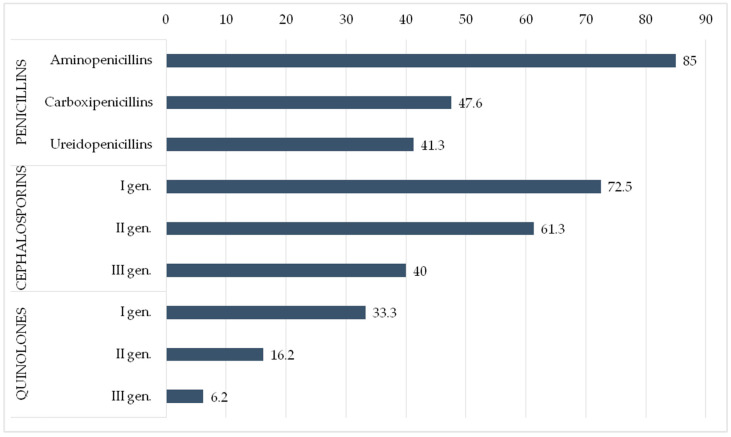
Percentage resistance to the different classes of penicillins, cephalosporins, and quinolones.

Finally, considering penicillins, cephalosporins, and quinolones, we further stratified the resistance results according to the different classes (Figure 6).

## 4. Discussion

Recent scientific evidence has shown how wastewater from slaughterhouses could be a source of antimicrobial-resistant bacteria with clinical importance and may thus play a role in their dissemination into the environment [33,34,35].

Many slaughtering activities are sources of contamination and release of microorganisms into the slaughter environment, among which defeathering and evisceration are considered the most important ones, mainly due to the discharge of intestinal content that occurs during these activities [36,37]. Especially for poultry slaughterhouses, previous studies have highlighted that the highest bacterial load is present in the first step of the entire process, the “hanging of poultry”, and then decreases during the defeathering process, before increasing again after evisceration. Finally, after the cooling process, the bacterial loads decrease again to the allowed CFU/g [36]. The problem of AMR in animal husbandry is mainly due to the shared use of many antimicrobials in both veterinary and human medicine. These resistant bacteria may not only be released into the environment from wastewater treatment plants (WWTPs), but they can also be transmitted to occupationally exposed slaughterhouse employees [38]. Following discharge into the environment through feces and wastewater, these strains are highly present in soil, plants, and surface water and may thus pose a risk for the colonization of humans [39,40], pets [41], and livestock [42].

Due to the high numbers of slaughtered animals, waters from slaughterhouses can be contaminated by ESKAPE bacteria that can be discharged following insufficient treatment in their in-house WWTPs [43,44,45], as recently reported for colistin-resistant, carbapenem-resistant, and extremely drug-resistant (XDR) bacteria in communal, hospital, and urban German wastewater [21,22,33].

In our study, we analyzed wastewater collected from slaughterhouses located in the Italian Region of Sicily. To our knowledge, this is the first report considering the detection of antibiotic-resistant bacteria in these kinds of samples in Italy. Among the bacterial genera, *Pseudomonas* spp. was the most frequently detected one, probably following its wide environmental diffusion, followed by enterobacteria belonging to the genera *Proteus* spp. and *Enterobacter* spp. A particular finding is the high detection rate of *Aeromonas* spp., an oxidase-positive, facultative anaerobic Gram-negative microorganism that commonly lives in aquatic environments and that was recently detected in slaughterhouse wastewater and in food such as meat and meat products and vegetables. *A. hydrophila* is able to affect humans, causing several diseases, especially gastroenteritis wound and soft tissue infections. *Aeromonas* spp. is especially considered an opportunistic pathogen. There is evidence of particularly virulent strains able to cause muscle infections, skin diseases, eye infections, pneumonia, and severe septicemia in immunocompetent patients [46]. Moreover, Saavedra et al. [47] stated that *A. hydrophila* causes hemorrhagic septicemia in stressed fish or those suffering from other illnesses, then representing a health risk to consumers. Multi-resistant *A. hydrophila* were isolated from different parts of the world and are reported to be resistant to penicillin and ampicillin but sensitive to aminoglycosides, tetracycline, chloramphenicol, trimethoprim-sulfamethoxazole, quinolones, and second- and third-generation cephalosporins. Moreover, in this strain, the increase in antibiotic resistance was documented and is now considered an important public health concern [48,49]. Less detected were *Citrobacter* spp. and *E. coli,* which together represent just over a tenth of all the detected strains.

Concerning antibiotic susceptibility, the most resistant strains were on average those belonging to *Enterobacter* spp., while the others showed mean moderately resistant levels. Specifically, *Enterobacter* spp. showed the highest resistance values to several antibiotic classes (cephalosporins, fosfomycin, monobactams, penicillins, quinolones, tetracycline) compared to the other detected genera. A moderately high resistance value to carbapenems was also detected. Moreover, *Aeromonas* spp., despite showing the lowest mean resistance value compared to the other detected genera, reported some important drawbacks such as a higher resistance value to carbapenems, second only to *Pseudomonas* spp. An important resistance to colistine was also detected. Finally, even if less detected, *E. coli* showed the highest resistance value to colistine, but no resistance was detected to carbapenems. However, this species, along with *Citrobacter* spp., showed complete resistance to macrolides, rifampicin, and tetracycline.

Concerning the tested drugs, quite high resistance levels were detected from some important antibiotic classes widely used in human medicine such as macrolides, penicillins, and cephalosporins. Specifically, the highest resistance rate was recorded for macrolides. Among β-lactams, penicillins, and cephalosporins were by far the molecules to which the highest resistance levels were detected, while a very low rate was recorded for carbapenems. Specifically, the highest resistance was detected for the longest molecules used, such as aminopenicillins and I generation cephalosporins. In particular, *Pseudomonas* spp. showed the highest resistance levels to β-lactams penicillins and cephalosporins but also showed the highest detected resistance to carbapenems. Additionally, for quinolones, towards which the strains showed an overall quite low resistance level, the highest resistance was detected for the longest used drugs. Colistine showed an overall moderate resistance rate, much lower, however, than that recorded in other settings. Indeed, Savin et al. [33] detected a percentage of colistine-resistant strains of 55.1% in German poultry and pig slaughterhouses. Finally, high resistance levels were found for tigecycline, tetracyclines, and rifampicin, while the lowest rate was recorded for aminoglycosides.

A very interesting finding is the difference in resistance levels reported in strains detected from poultry slaughterhouses and those revealed in strains originating from slaughterhouses butchering only ungulates (bovine, pig and sheep farms). Indeed, in the first we detected higher levels for almost all the considered drugs, with remarkable differences especially for aminoglycosides, fosfomycin, macrolides, quinolones and tetracyclines. Probably, this result is a consequence of the massive use of antibiotics present in poultry farms compared to that in ungulates. In recent decades, broilers have become one of the most relevant meat source with United States, Brazil, China, and some countries of the European Union (Poland, United Kingdom, Germany, France, and Spain) as the major world producers accounting for approximately 60% of the total worldwide production. Antibiotics in poultry are used not only for the treatment of disease (therapeutic purpose) but also for prevention (methaphylactic purpose), and even for growth promotion (auxinic purpose). Moreover, these drugs are generally administered to entire flocks [50,51]. In these farms, antibiotics are used for the treatment of some intestinal infections such as colibacillosis, necrotic enteritis, and other diseases commonly caused by *Salmonella*, *E. coli*, or *Clostridium* spp. The use to this purpose is due to the fact that these infections are an important concern among poultry producers leading to enormous economic losses [52].

It has been shown that there is a continuous and reciprocal exchange of microorganisms between animal, environment, and man. This can occur through direct human–animal contact, food of animal origin, and wastewater dispersed in the environment. For this reason, the phenomenon of antibiotic resistance is a problem that must be addressed in a One Health vision, since human and animal health on one hand and the environment on the other are strongly interconnected. The concept of One Health encourages collaboration across different professions and sectors to reach a more holistic vision of the health concerns and threats addressing the efforts at the human–animal-environment interface. This approach has more and more been adopted at national and international levels, becoming an integral part of plans and strategies for the management of zoonoses, health security, food safety, veterinary medical education, and antimicrobial resistance [53].

To date, it is not known what the real impact is of antibiotic resistance of zootechnical origin, but it is important to strongly recommend that veterinarians and breeders engage in the rational and prudent use of antibiotics in animals. The production of more and more food requires a large-scale production of safe and quality products. However, although the use of antibiotics for auxinic purposes has been banned in animal husbandry since 2006 in the EU [54] and 2017 in the US [55], even today the use of antibiotics is not always appropriate. Antibiotic use for methaphylactic purposes is allowed in all large poultry-producing countries, and the auxinic use is currently permitted Brazil and China [55]. Therefore, in order to contain antimicrobial resistance, the scientific world agrees that antibiotics must be used in compliance with the indications for use, only for therapeutic purposes and following a specific diagnosis, while the use for prophylactic and metaphylactic purposes should be avoided or however severely limited [56]. Specifically, since 2005, WHO has published a periodically revised list of critically important antimicrobials for human medicine to be used cautiously. In this list, all antibiotics currently used both in humans and in animals are grouped into three categories, “important”, “highly important”, and “critically important”, based on their significance in human medicine. The aim is to encourage cautious use to decrease the spread of antimicrobial resistance and preserve the efficacy of the most critical antibiotics for human medicine. In the sixth revision of the list published in 2018 [57], the antibiotics considered highest priority critically important antimicrobials are quinolones, third and higher generation cephalosporins, macrolides and ketolides, glycopeptides, and polymyxins (also known as colistin) because these drugs are essential as last-resort treatments for multidrug-resistant infections in humans.

## 5. Conclusions

Our results indicate slaughterhouse wastewater as a potential vehicle of antibiotic-resistant bacteria and therefore a possible environmental contamination route. Their role in the spread of antibiotic resistance could be of primary importance also considering the high number of slaughterhouses present on the Italian territory. In particular, some critical findings such as the presence of resistance to carbapenems and colistine (now considered key drugs in human medicine for the treatment of severe multidrug-resistant infections) were reported. For this reason, considering the huge importance that antimicrobial resistance plays nowadays in human medicine and the widespread production of this kind of water, we intend to evaluate this concern by analyzing wastewater from different settings. We have already started the research into multidrug-resistant bacteria in wastewater from dairies, and soon we will include wastewater from aquaculture facilities. Therefore, in order to contain the environmental risk of cross-contamination, it is crucial to carry out correct management of this kind of water, especially in the treatment phase. Finally, in order to prevent the onset and diffusion of antibiotic resistance in this setting, strict regulation and continuous monitoring on the correct use of antibiotics in animal husbandry according to the already in place legislation are necessary.

## 6. Limits of the Study

This study is a general phenotypic overview of the presence of antimicrobial-resistant bacteria in a specific material represented by wastewater from slaughterhouses. Genetic characterization of antimicrobial resistance was not carried out because it will be the focus of future investigations already in place. Moreover, the analysis was carried out on wastewater samples before they underwent any treatment. Even this aspect will be the object of future investigations in which treated wastewater samples will include in order to evaluate the difference with the untreated ones.

## Figures and Tables

**Table 1:** Percentage resistance levels of the detected genera to the antibiotic families used.

	*Aeromonas* spp.	*Citrobacter* spp.	*Enterobacter* spp.	*E. coli*	*Proteus* spp.	*Pseudomonas* spp.
Aminoglycosides	7.2	0.0	9.4	0.0	2.2	5.5
Carbapenems	14.3	0.0	12.5	0.0	0.0	18.2
Cephalosporins	28.6	33.3	75.0	50.0	48.1	60.6
Cloramphenicol	0.0	0.0	12.5	0.0	33.3	45.5
Colistine	28.6	33.3	0.0	50.0	33.3	9.1
Fosfomycin	57.1	0.0	75.0	50.0	33.3	18.2
Macrolides	71.4	100.0	87.5	100.0	88.9	63.6
Monobactams	28.6	33.3	75.0	0.0	33.3	33.3
Nitrofurans	57.1	66.7	87.5	100.0	66.7	63.6
Penicillins	40.0	33.3	72.0	50.0	28.5	68.9
Quinolones	11.4	6.7	32.5	12.5	22.2	19.2
Rifampicin	42.9	100.0	87.5	100.0	33.3	54.5
Tetracyclines	71.4	33.3	87.5	50.0	55.6	54.5
Tigecycline	57.1	100.0	87.5	100.0	77.8	54.5
Mean resistance value	41.0	42.7	60.1	50.8	43.8	45.4

## Data Availability

Not applicable.

## Supplementary Materials

The following are available online at https://www.mdpi.com/article/10.3390/ijerph18189611/s1, Table S1: List of antibiotics used to determine the antibiotic resistance patterns of the bacterial isolates.
