# Supplementary material for: Evaluation of Antibiotic Resistance in Bacterial Strains Isolated from Sewage of Slaughterhouses Located in Sicily (Italy)
Source: Int J Environ Res Public Health. 2021 Sep 12;18(18):9611. doi: 10.3390/ijerph18189611 (PMC8467622; doi:10.3390/ijerph18189611)
Supplement: Supplementary file 1 [file ijerph-18-09611-s001.zip › ijerph-1360356-supplementary.pdf]

International Journal of
Environmental Research and Public Health

MDPI

Supplementary Material for “Role of wastewater from slaughterhouses in the spread of antibiotic resistance” Facciolà A, Virga A, Gioffrè ME, Laganà P.

Table S1. List of assayed antibiotics with the doses for each drug, grouped in classes according to their mechanism of action.

CELL WALL INHIBITING AND DISRUPTING MEMBRANE ANTIBIOTICS	**B-LACTAMS**	**PENICILLINS**	*Natural penicillins*	*penicillin (P, 1 unit,)*
			Aminopenicillins	*amoxicillin (AML, 10 μg)*
				ampicillin (AMP, 10 μg)
			Carboxipenicillins	*carbenicillin (CAR, 100 μg)*
			Ureidopenicillins	*mezlocillin (MEZ, 75 μg)*
				piperacillin (PRL, 100 μg)
			Penicillinase-resistant penicillins	*oxacillin (OX, 1 μg)*
				meticillin (MET, 5μg)
			Combinated penicillins	*amoxicillin + clavulanic acid (AMC, 30 μg)*
		**CEPHALOSPORINS**	*1st generation*	*cefazolin (KZ, 30 μg)*
			2nd generation	*cefoxitin (FOX, 30 μg)*
				cefuroxime (CXM, 30 μg)
			3rd generation	*cefotaxime (CTX, 30 μg)*
				ceftazidime (CAZ, 30 μg)
				ceftriaxone (CRO, 30 μg)
		**CARBAPENEMS**	*imipenem* (IMI, 10 μg)	
		**MONOBACTAMS**	*aztreonam* (AZM, 30 μg)	
	**GLYCO - PEPTIDES**	*vancomycin (VAN, 30 μg)*		
		teicoplanin (TEC), 30 μg)		
	fosfomycin (FOS, 50 μg)			
	**POLYMIXINS**	*colistin sulphate* (CS, 10 μg)		
NUCLEIC ACIDS INHIBITING ANTIBIOTICS	**INHIBITING DNA TOPOISOMERASIS ANTIBIOTICS**	**QUINOLONES**		*cinoxacin (CIN, 100 μg)*
				nalidixic acid (NA, 30 μg)
				pipemidic acid (PI, 20 μg)
		**FLUORQUINOLONES**		*ciprofloxacin (CIP, 5 μg)*
				levofloxacin (LEV, 5 μg)
				norfloxacin (NOR, 10 μg)
				ofloxacin (OFX, 5 μg)
	**INHIBITING FOLIC ACID**	**SULFONAMIDES**		*sulphamethoxazole + trimethoprim*

	**SYNTHESIS ANTIBIOTICS**		(SXT, 25 µg)
	**INHIBITING RNA SYNTHESIS ANTIBIOTICS**	**RIFAMYCINS**	*rifampicin* (RD, 30 µg)
	**DNA INHIBITORS ANTIBIOTICS**	**NITROFURANS**	*nitrofurantoin* (F, 300 µg)
**PROTEIN SYNTHESIS INHIBITING ANTIBIOTICS**	**30S SUBUNIT INHIBITORS**	**AMINOGLYCOSIDES**	*amikacin* (AK, 30 µg)
			gentamycin (CN, 10 µg)
			netilmicin (NET, 30 µg)
			sisomicin (SIS, 30 µg)
			tobramycin (TOB, 10 µg)
		**TETRACYCLINES**	*doxycyclin* (DO, 30 µg)
			tetracycline (TE, 30 µg)
			minocycline (MN, 30µg)
		**GLYCYLCYCLINES**	*tigecycline (TGC, 15 µg)*
	**50S SUBUNIT INHIBITORS**	**MACROLIDES**	*azithromycin* (AZM, 15 µg)
			eritromycin (E, 15 µg)
		**LINCOSAMIDES**	*lincomycin* (MY, 2 µg)
			clindamycin (DA,10 µg)
		**OXAZOLIDINONES**	*linezolid* (LNZ, 10 µg)
		**PHENOLIC DERIVATIVES**	*chloramphenicol* (C, 30 µg)
